# Study on the biodegradability of modified starch/polylactic acid (PLA) composite materials

**DOI:** 10.1039/d0ra00274g

**Published:** 2020-07-13

**Authors:** Meihong Yu, Yongjie Zheng, Jingzhi Tian

**Affiliations:** College of Chemistry and Chemical Engineering, Qiqihar University Qiqihar 161006 Heilongjiang China zyj1964@163.com

## Abstract

In this work, polylactic acid/thermoplastic acetylated starch (PLA/TPAS) composites were prepared using PLA as a matrix material and TPAS as a modifier. TPAS is based on acetylated starch, which is plasticized using glycerin. Analysis of the mechanical, thermal, and dynamic mechanical properties, and morphological structures of the PLA/TPAS composites shows that with an increase in the TPAS content, the toughness of the PLA/TPAS composites significantly improves. When the amount of TPAS added is 40% by weight, the elongation at break is increased 4 times. At the same time, the addition of TPAS has little effect on the thermal stability of the composites. Differential scanning calorimetry (DSC), dynamic mechanical analysis and scanning electron microscopy (SEM) analysis results show that PLA is incompatible with TPAS. The addition of TPAS promotes the crystallization of PLA, resulting in a decrease in the thermal stability but limits the degradation behavior during the processing of the material, which has little effect on the performance of the material. High temperature and high humidity soil degradation and ultraviolet radiation aging experiments on PLA/TPAS composites show that the PLA/TPAS composites have good biodegradability. In soil burial degradation experiments, the degradation rate of the pure PLA material is slow, and its final mass retention rate is high. The PLA/TPAS composites degrade fast. In ultraviolet radiation aging experiments, the tensile strength of the PLA/TPAS composites was improved to a certain extent after exposure to ultraviolet radiation. With an increase in the ultraviolet irradiation time, the tensile properties of the PLA/TPAS composites gradually decreased.

## Abstract 2

In this work, polylactic acid/thermoplastic acetylated starch (PLA/TPAS) composites were prepared using PLA as a matrix material and TPAS as a modifier.

## Graphical abstract

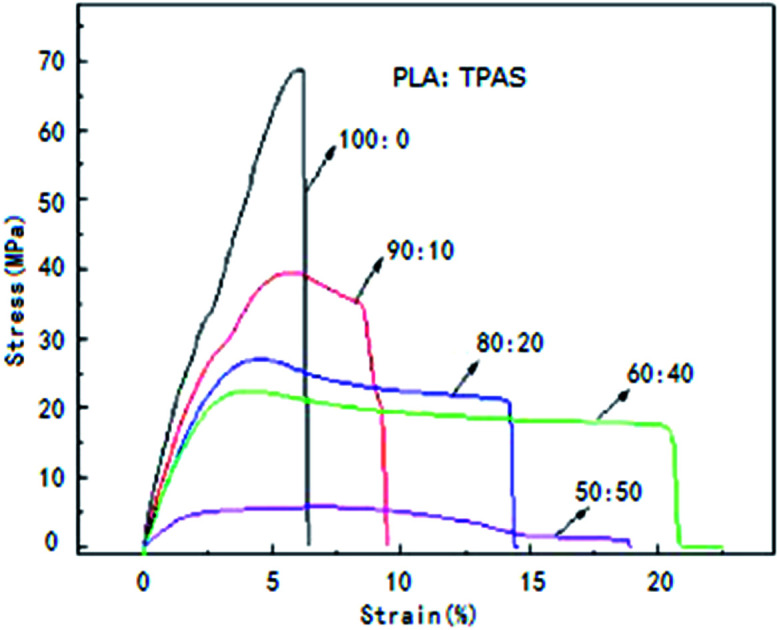

## 1 Introduction

Biocomposite materials are composed of two or more different materials, and generally have good biocompatibility, biological stability, certain strength and toughness, and good antibacterial properties.^1,2^ Polylactic acid (PLA) has good biocompatibility, safety, and biodegradability. It eventually decomposes into CO_2_ and H_2_O in the environment, producing no other environmental pollutants. It is approved by the FDA (US Food and Drug Administration) as being biodegradable. Materials widely used in the fields of surgical sutures, orthopedic fixation materials, *in vivo* implant materials, and medicinal control systems, are globally recognized as the most promising biomedical materials this century.^3^ PLA is produced using lactic acid as a raw material. Most traditional fermentation processes use starchy raw materials to produce lactic acid. So far the United States, France, and Japan have developed agricultural and sideline products as raw materials to produce lactic acid. Polymerization is then carried out to produce PLA.

Starch is abundant, cheap, and easily biodegrades. It is usually found in pellets in a large number of plants such as wheat rice and corn. Biodegradable polyester and cheap starch composite systems with excellent performance are materials that can be used to resolve the discrepancy between the performance and cost.^4–6^ However, natural starch has a rigid granular structure with strong hydrogen bonds between molecular chains and no thermoplasticity. Therefore, in this study, by adding the plasticizer glycerin, we show how the hydrogen bonds between starch molecules can be weakened, improving the molecular chain diffusion and reducing the glass transition temperature, therefore producing microcrystals that melt before their decomposition. The starch matrix used this time is acetylated modified starch. The acetylated starch has acetyl groups, which weaken the intermolecular hydrogen bonds.^7,8^

PLA can be modified in a number of ways. After it is copolymerized and modified, the crystallinity of PLA can be improved. Copolymerized modified PLA has improved flexibility and elasticity, effectively improved mechanical properties and reaction functionality, and its degradation cycle and hydrophilic and lipophilic properties are changed. Most copolymerized modified products of PLA are biodegradable materials.^9^ In 1997, Ogata first reported PLA nanocomposites, and found that the addition of montmorillonite improved the crystallization properties and Young's modulus of the PLA. Paul *et al.* successfully produced three exfoliated PLA/montmorillonite nanocomposites using lactide as a monomer *via in situ* intercalation polymerization.^10^ The results showed that the montmorillonite sheets in the composite material prepared using montmorillonite containing hydroxyl groups had a better peeling effect than the composite material prepared using montmorillonite without any hydroxyl groups.

In this paper, PLA/TPAS (TPAS = thermoplastic acetylated starch) composites were prepared by melt blending. By controlling the composition ratio of the blend and using TPAS to plasticize PLA, we intended to both improve the performance of the material and reduce the price of the material, so as to obtain a fully biodegradable composite material that has excellent performance and low cost.

### 1.1 Research on PLA modification

PLA has some excellent properties, such as higher mechanical strength, it is easy to process, and has better ductility, but it is brittle, has poor toughness, low elongation at break, lacks flexibility and elasticity, and has a low flexural modulus. These characteristics limit the application of PLA in the field of packaging, agricultural mulching film, and fresh-keeping materials.^11,12^ Therefore, PLA must be softened and toughened. At present, the modification methods for PLA softening and toughening mainly include copolymerization, composite, and blending modification processes.

In the study of copolymerization modification, Wang *et al.* polymerized lactide with polyethylene glycol (PEG) to obtain low copolymer PLLA (poly(l-lactide))–PEG.^13^ After secondary polymerization, PLLA–PEG copolymer was formed and then lysine was used to modify the PLLA–PEG copolymer. Differential scanning calorimetry (DSC) tests showed that the melting and melting point of the modified PLLA–PEG copolymer were reduced, from 81.57 J g^−1^ and 177.34 °C to 46.02 J g^−1^ and 151.34 °C, respectively, which effectively improved the molecular chain flexibility and crystalline properties. Water contact angle tests showed that the lysine-modified PLLA–PEG copolymer has good water solubility, and its water contact angle is 67°, while the pure PLLA and PLLA–PEG copolymers have water contact angles of 114° and 82°.^14^ It has therefore been proven that the addition of hydrophilic lysine improves the hydrophilicity of the PLLA–PEG copolymer and is a good bioengineering material.

In a study on blend modification, Carbonell *et al.* used maleicated cottonseed oil (MCSO) and maleated linseed oil (MLO) as plasticizers to modify PLA films,^15^ and compared and analyzed the mechanical, thermal, and barrier properties, and morphological changes of the modified PLA films. The results show that the crystallinity of the PLA/MCAO films is lower than that of the PLA/MLO films, but that PLA/MCAO films are better than PLA/MLO films in terms of mechanical properties. Adding 7.5% MCAO made the elongation at break of the film reach 292%. After the addition of MCAO and MLO, the glass transition temperature of the PLA film decreased by about 2 °C, while the melting temperature did not change much. PLA/MCAO films are superior to PLA/MLO films in terms of barrier properties. The study concluded that MCAO plasticized PLA film is better than MLO and is a good plasticizer for modified PLA.

In their research on composite modification, Arrieta *et al.* studied the structure and properties of PLAIPHB (poly light butyrate)/ATBC/CNC (nanocellulose) degradable nanocomposites.^16^ A 25% increase in the weight ratio of PHB and PLA made the material more uniform and it did not lead to any obvious bead fibers being produced during the electrospinning process. When the ATBC content is 15% by weight, the glass transition temperature of the composite material decreases and the elongation at break significantly increases. The addition of 1 wt% CNC helps to improve the thermal stability, crystallinity and mechanical strength of the composite. Pluta *et al.* studied the effects of kaolin nanotubes (HNT) and acetylene bisarylamide (EBS) on the cold crystallization temperature, dynamic mechanical and optical properties of PLA/JHNTIEBS composites, and concluded that the nucleation of HNT can reduce the cold crystallization temperature of lactic acid from 111.7 to 103.6 °C.^17^ NNT enhances the storage modulus of PLA, and the light transmittance is reduced by HNT dispersed in the PLA matrix, but NHT easily aggregates in the PLA matrix to form fine particles, which affects the performance of the composite.

Among the many methods for modifying PLA films, plastic modification and composite modification are the more commonly used modification methods. Although plasticized modification reduces the high strength and high modulus of PLA and also reduces its melt strength, it does not affect the transparency and processing properties of PLA films. At the same time, plastic modification can also significantly improve the tensile properties and flexibility of PLA films. Composite modified PLA films have improved heat resistance and mechanical properties. However, the effect of composite modification is not as good as plastic modification. After the PLA film is compositely modified, phase separation is likely to occur, and the transparency of the modified PLA film is also reduced.^18,19^

### 1.2 Experimental research on the degradation of PLA composites

PLA is a completely environmentally friendly, degradable polycool material. No matter how lactic acid is polymerized, the essence of the forming of PLA is by the dehydration of lactic acid *via* ester bond polycondensation.^20^ The ester bond can be broken under many conditions, so PLA has multiple degradation pathways, such as hydrolysis degradation, microorganism degradation and UV (ultra-violet) degradation.

The nature of PLA and the environmental conditions of hydrolysis determine the mechanism, rate and process of its hydrolytic degradation. The higher the crystallinity and molecular stereoregularity of PLA, the less likely it is to degrade. For semi-crystalline PLA, the hydrolytic degradation process is from the amorphous to crystalline region. When the hydrolysis temperature is higher than the glass transition temperature (*T*_g_), the rate of hydrolysis of PLA will be significantly increased. If the hydrolysis temperature is higher than the melting temperature (*T*_m_), the hydrolysis degradation mechanism of PLA will change, and the PLA crystal zone melts and disappears, and homogeneous hydrolysis easily occurs in the molten zone.^21^

Roccasmith *et al.* studied the chemical stability and hydrolysis mechanism of PLA films under high temperature and high humidity conditions.^22^ The results show that the chemical properties of PLA films are stable under the conditions of 50% RH (relative humidity) and a temperature of 50 °C. They were characterized by their appearance, microstructure, surface hydrophobicity, and the number of amorphous and crystalline regions they contained. The *T*_m_ and *T*_c_ did not change significantly, indicating that there was no water degradation during the entire degradation process, but that physical aging occurred because the film was stored at *T*_g_ temperature for a long time. At 100% RH or in a completely aqueous environment, the degradation of the PLA film changes significantly, the transparency of the film decreases, and the microstructure changes. At the same time, the *T*_g_, *T*_m_, *T*_c_ and other thermal parameters of the film are reduced; the degradation products released by the PLA film in a completely aqueous solution (lactic acid, small molecule plasticizer) will become a catalyst to accelerate its water degradation process; so the degradation rate of the PLA film will be accelerated at high temperature under high humidity conditions, which will reduce its performance and render it less useful.^23–26^

Ramos *et al.* prepared binary and ternary PLA active nanocomposite films using PLA as the matrix material, vanillin and AgNPs (silver nanoparticles) as fillers.^27^ For the PLA-based active nanocomposite films, through the carrying out of DSC and thermogravimetric (TG) tests, it was found that after adding thymol and AgNPs, the *T*_g_ and *T*_m_ of the PLA decreased, and therefore, the thermal stability of the PLA-based composite film also decreased. Studies of degraded PLA-based active nanocomposite films have shown that the addition of vanillin and AgNPs can accelerate the degradation rate of PLA composite films. The synergistic effect of the vanillin and Ag-NP PLA composite films is obvious.

Hai *et al.* prepared PLA/CS (chitosan) degradable composite films using a non-solvent induced phase separation.^28^ From the scanning electron microscopy (SEM) image and the light transmission of the film, it was found that the composite film has a porous structure and high transparency, and the light transmittance exceeds 95%; the thermal stability of the composite film was found to be reduced upon the carrying out of TG and DSC tests, and water contact angle tests were used to verify the hydrophilicity of the composite membrane. After testing, the composite membrane was found to have a bacteriostatic rate of 99.77%.^29–31^ In experiments on the degradation of PLA/CS composite membranes, researchers found that the degradation rate is directly proportional to the pore size. For a composite with six parts PLA and one part CS, the degradation rate of the PLA/CS composite membrane was found to be the lowest. This shows that the composite membrane has great application potential for use in biomedicine, food packaging materials and adsorbent materials.

In UV degradation tests, PLA can undergo degradation reactions under the promotion of invisible low-wavelength light and high-energy UV.^32^ PLA molecules undergo chemical reactions such as main chain cleavage, bond cleavage, chain cross-linking, and oxidation after absorbing UV light. On the macro level, PLA materials exhibit discoloration, deformation, and brittle fracture.^33^

The degradation experiments of PLA-based composite materials are mostly performed using a soil buried method at high temperature and under high humidity conditions and PLA-based composite materials are subjected to aging experiments in an ultraviolet aging box.^34^ Laboratory soil buried high temperature and high humidity degradation and UV box aging experiments can provide some theoretical basis and research methods for the degradation of PLA materials under natural conditions.

## 2 Experimental

### 2.1 Materials and methods

See Tables 1 and 2.

**Table 1:** Experimental equipment and testing instruments

Equipment	Model	Factory
Torque rheometer	Haake	Thermo Haake Polylab System
Flat curing machine	QLB-D600 × 600	Jinxiang Rubber Plastic Machinery Factory
Differential scanning calorimeter	PerkinElmer DSC-7	PerkinElmer Asia
Universal material testing machine	WSM-20KN, GB/T1040.1-2006	USA Instron
Izod impact tester	UJ-40	Wuzhong Material Testing Machine Factory
Thermal gravimeter	PerkinElmer TGA-7	PerkinElmer Asia
Synchronous thermal analyzer	STA449F3	Netzsch, Germany
Dynamic mechanics analyzer	DMA-242C	Netzsch, Germany
Scanning electron microscope	JAK-840	JEOL, Japan
UV aging lamp	60 W	Self-made

**Table 2:** Experimental main raw materials and reagents

Name	Properties	Supplier
Polylactic acid (PLA)	2003D, *M*_w_ = 1.77 × 10^6^, polydispersity coefficient *n* = 1.43	NatureWorks, USA
Acetylated starch	Degree of acetyl substitution 1.0–1.5	Shandong Zhucheng Xingmao Corn Development Co. Ltd
Glycerin	Analytically pure	Beijing Chemical Plant
Humus soil	—	Collected from outside at Qiqihar University

### 2.2 Preparation of thermoplastic acetylated starch (TPAS)

The current methods for preparing TPAS include *in situ* polymerization, phase separation, electrostatic spinning, surface coating, and melt blending. The melt blending method has the advantage of simple operation, and inorganic filler particles can be incorporated into the polymer matrix using mechanical force. The advantage of the melt blending method is that the morphology and size of the material particles can be controlled during the preparation process. Therefore, the melt blending method was used to prepare the TPAS in this work.

The acetylated starch and glycerin were pre-mixed in a mass ratio of 60 : 40, and sealed together in a vessel and stored for 24 h. The pre-mix was melt-blended on a Haake (Germany) torque rheometer. The blending conditions were a temperature of 150 °C and a rotation speed of 60 rpm, with a mixing time of 10 min, to obtain thermoplastic acetylated starch (TPAS) for future use.

### 2.3 Preparation of PLA/TPAS

PLA and TPAS were melt-mixed on a Haake (Germany) torque rheometer. The mixing temperature used was 180 °C, the rotation speed was 50 rpm, and the mixing time was 6 min. The temperature, rotation speed and mixing time were kept the same throughout the experiment, and PLA/TPAS composite materials with different ratios were prepared. At the same time, the blended samples were cut into small pieces, and heated at 180 °C and 10 MPa on a flat plate curing machine. After hot pressing for 5 minutes, the samples were pressed into thin sheets, and then placed in a cold press to maintain the pressure and allow them to cool to room temperature for setting to occur.

### 2.4 Testing and measurement methods

Thermal performance tests were carried out on a PerkinElmer DSC-7 differential scanning calorimeter. The sample amount was about 10 mg, and the heating rate is 10 °C min^−1^ under a nitrogen atmosphere.

Thermogravimetric analysis measurements were recorded on a PerkinElmer TGA-7 thermogravimetric analyzer at a heating rate of 2 °C min^−1^, and a temperature range of 40–540 °C.

For the tensile properties, data were measured on an Instron model 1211 universal materials testing machine. The spline was a dumbbell shape of 50 mm × 3.5 mm × 1 mm in size, and the test standard used was GB/T1040-1992. Five splines were tested and averaged. The test temperature was 25 °C and the tensile rate was 15 mm min^−1^.

For the impact performance, data were measured using a UJ-40 cantilever impact tester. The samples were made into rectangular splines of 63.5 mm × 12.7 mm × 3.2 mm in size using a sample cutting machine, and a V-shaped notch was milled on a notch machine to obtain a cantilever beam notch impact spline, and then five splines were tested and averaged. The test standard used was GB/T1843-1996.

For the dynamic mechanical performance analysis, a DMA-242C type dynamic mechanical analyzer (Netzsch, Germany) was used to measure the temperature dependence of the storage modulus and loss factor (tan *δ*) of the sample. In stretching mode, the sample was first molded at 180 °C. The heating rate of the sample was 3 °C min^−1^, the frequency was 1 Hz, and the temperature range was −85–125 °C. The test is completed in a nitrogen atmosphere.

For the observation of the morphology of the composite material, after the impact test, the notched sample was sprayed with gold, and the cross-section morphology was observed using a JAK-840 scanning electron microscope (JEOL Corporation, Japan).

Degradation performance tests:

(1) Degradation experiment at high temperature with high humidity soil: first, four plastic foam insulation boxes were prepared, and 5 cm of humus soil was at the bottom of each plastic foam insulation box, and film samples with dimensions of 10 cm in length and 5 cm in width were spread neatly on the humus soil, then the films were covered with a further 5 cm thick layer of humus soil, a certain amount of deionized water was added, and finally, the four plastic foam insulation boxes were weighed and recorded, and the plastic foam boxes were kept at a constant temperature of 40 °C. The water in the boxes was weighed every 12 h, and the film quality was assessed by weighing them every 5 days. The weight was measured 5 times, and the weight retention rate was calculated after weighing the film each time.

(2) UV radiation aging experiment: the UV irradiation aging of the PLA-based composite film was measured using a self-made UV irradiation lamp (60 W) in a radiation box. The PLA-based composite film was cut into strips for tensile property testing. The strips were evenly arranged around the UV lamp. The distance between the sample and the UV lamp was 30 cm. The experimental temperature was about 25 °C. Samples were taken every 5 h. The tensile properties were measured. Five aging splines were tested in each group, and the test results were averaged.

## 3 Results and discussion

### 3.1 Mechanical properties of PLA/TPAS

**Fig. 1:**
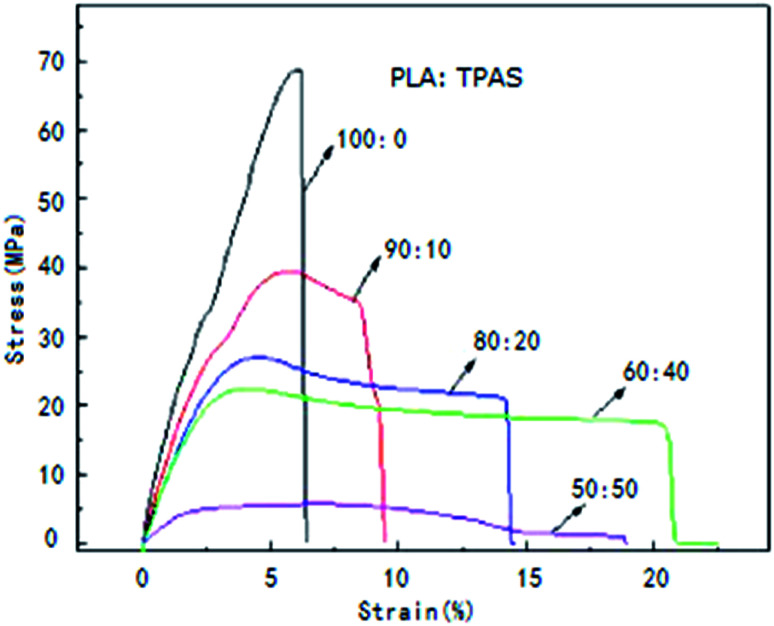
Stress–strain curves of the PLA/TPAS composites with different mass ratios.

To improve the mechanical properties of PLA, PLA was blended with TPAS. Fig. 1 shows the stress–strain curves of the PLA/TPAS composites. Pure PLA shows typical brittle fracture characteristics. Its elongation at break is low and there is no obvious stress yield before fracture. For composite materials, when the mass fraction of TPAS is 10%, the yield point is present in the stress–strain curve, but its elongation at break is low. As the content of TPAS increases further, the material exhibits the characteristics of ductile fracture. Obvious yielding inflection points and elastic deformation stress platforms appear in the strain curve. Composite materials also show obvious bottleneck shrinkage stress whitening during the stretching process. The tensile sections of the samples were rough and irregular.^35,36^ The results of toughness analysis show that adding TPAS can increase the toughness of PLA and improve its brittleness to a certain extent. When the amount of TPAS was 40% by weight, the tensile toughness of the composite was the best.

Table 3 shows the values of the impact strength, tensile strength, elongation at break, and elastic modulus of the PLA/TPAS composites as a function of composition. It can be seen that pure TPAS exhibits the characteristics of an elastomer, with lower tensile strength and higher elongation at break. With an increase in the TPAS content, the impact strength of the PLA/TPAS composites increases and the toughness increases. When the amount of TPAS added was 40%, the elongation at break was increased by more than 4 times. In the same way, glycerin was used as a plasticizer, and the impact strength of the corn starch/PLA composites did not increase significantly (only by 3%).^37^ This shows the high strength of PLA/TPAS, indicating that the mechanical properties of composites made from TPAS are superior. PLA is a brittle material with high modulus (1.7 GPa) and low elongation at break (5.8%). When the content of TPAS increases, the tensile modulus and tensile strength of the composite material decrease, and the elongation at break increases. When TPAS is added in an amount of 40% by weight, the composite material exhibits better comprehensive mechanical properties.

**Table 3:** Mechanical properties of the PLA/TPAS composites

TPAS : PLA	Impact strength (kJ m^−2^)	Tensile strength (MPa)	Elongation at break (%)	Tensile modulus (GPa)
0 : 100	5.2 (±0.3)	59.8 (±0.3)	5.8 (±0.3)	1731.8 (±32.5)
10 : 90	8.7 (±0.3)	43.4 (±0.1)	14.5 (±0.3)	1352.1 (±32.8)
20 : 80	9.1 (±0.7)	39.3 (±0.4)	22.6 (±0.3)	1227.8 (±12.9)
40 : 60	10.5 (±1.2)	24.6 (±0.3)	26.7 (±0.3)	978.1 (±24.6)
50 : 50	13.1 (±1.3)	14.8 (±0.2)	38.1 (±0.4)	711.1 (±16.4)
70 : 30	17.5 (±1.8)	7.1 (±0.1)	179.2 (±4.1)	54.6 (±0.4)
100 : 0	No break	2.7 (±0.3)	329.4 (±3.6)	4.2 (±0.1)

### 3.2 DSC analysis of PLA/TPAS

**Fig. 2:**
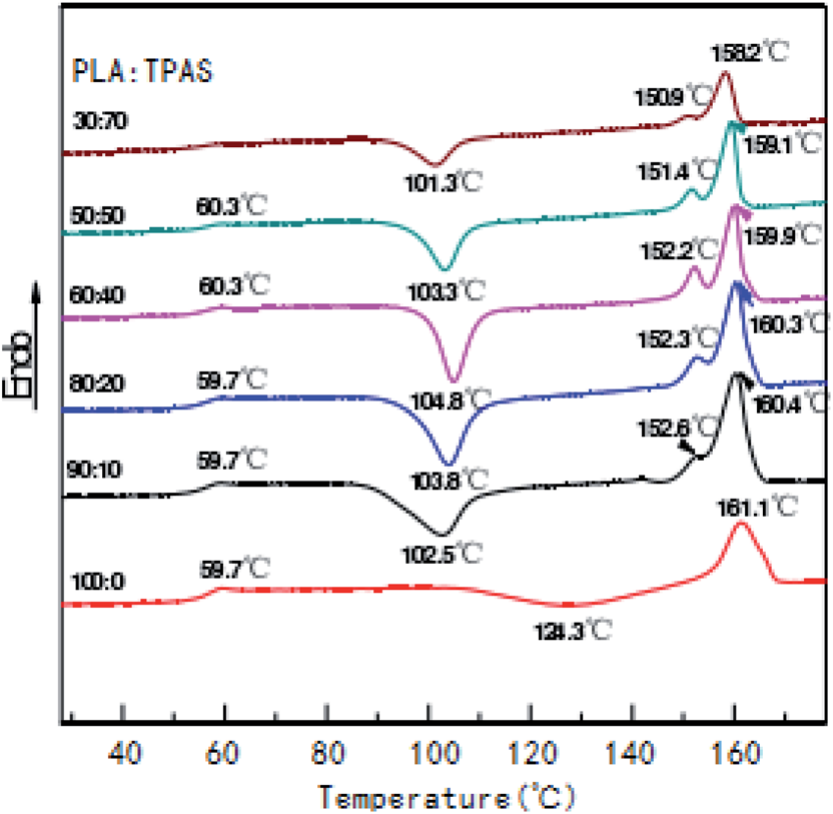
DSC curves of the PLA/TPAS composites with different mass ratios.

Fig. 2 shows the secondary heating curve of the PLA/TPAS composite materials after quenching at a heating rate of 10 °C min^−1^. Because starch is not sensitive to temperature, the graph only shows the thermal behavior of PLA. Pure PLA shows a glass transition at around 60 °C. When the content of TPAS increases, the glass transition of PLA decreases. When the TPAS content reaches 70% by weight, the glassy state basically disappears. This shows that TPAS is incompatible with PLA. Near 103 °C, the composites with TPAS showed significant cold crystallization, while pure PLA did not experience cold crystallization at this temperature. This shows that the addition of TPAS reduces the cold crystallization temperature of PLA. With an increase in the TPAS content, the compatibility of TPAS and PLA is improved. On the other hand, after adding TPAS, the cold crystallization and melting behavior of PLA changed significantly. There are two melting peaks in the data for PLA/TPAS. This shows that there is phase separation between PLA and TPAS, and their compatibility is not very good. Pure PLA shows a clear and broad cold crystallization peak near to 124 °C. When the content of TPAS increases, the cold crystallization peak of PLA gradually moves towards low temperature, and its peak shape becomes significantly narrower. This shows that the addition of TPAS significantly accelerates the crystallization of PLA, especially for PLA/TPAS (40 : 60) composites. This may be due to TPAS acting as a nucleating agent during PLA crystallization. At the same time, the small molecules of glycerol reduce the interaction between the PLA molecules, which reduces the barriers to be overcome when the molecules are regularly arranged, so obvious crystallization occurs. The crystallization rate of pure PLA is relatively slow, which limits the maintenance and improvement of its performance during certain processes.^38–40^ The addition of TPAS provides the possibility to effectively control the crystallization rate and has certain industrial production significance.

### 3.3 DMTA analysis of PLA/TPAS

**Fig. 3:**
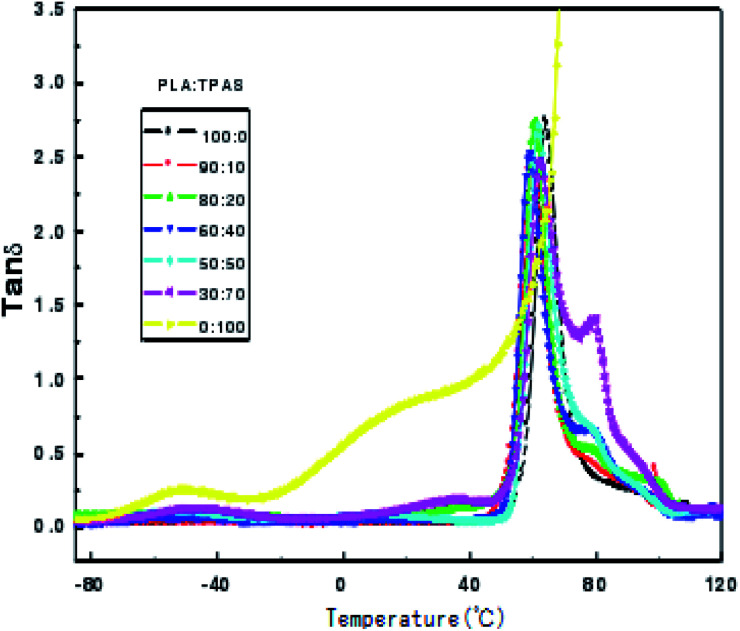
Plots of tan *δ versus* temperature for the PLA/TPAS composites with different mass ratios.

Dynamic mechanical analysis is the analysis of changes in the strain and stress relationship with temperature and other conditions under the action of external forces. Dynamic mechanical analysis can be used to obtain the polymer's dynamic modulus, loss modulus and mechanical loss. These physical quantities are important parameters that determine the use characteristics of polymers. Fig. 3 shows the change curve of the loss tangent tan *δ* of the PLA/TPAS composite materials with temperature *T*. It can be seen that TPAS shows transition peaks at around −50 and 36 °C, respectively. The transition zone at near to −50 °C corresponds to a phase composed of water and glycerol in which a small amount of acetylated starch molecules are dissolved, which is the glass transition of a glycerol-rich phase. The transition zone near to 36 °C corresponds to the glass transition of the acetylated starch-rich phase with the amorphous phase of glycerol and water as plasticizers and a large number of starch molecules.^41^ The glass transition peak of PLA is at around 60 °C. Loss peaks corresponding to the two phases are in the data for the composite material, and the peak positions of the loss peaks show no significant change. This shows that the PLA and TPAS phases in the PLA system are completely incompatible. This is also consistent with the conclusions drawn from DSC analysis. As the amount of TPAS added changes, the loss tangent tan *δ* peak intensity at the glass transition temperature of PLA changes. This may be due to the change in the amount of glycerol when different amounts of TPAS are added to PLA.

### 3.4 SEM analysis of PLA/TPAS

In order to investigate the microstructure of the composite material, we performed SEM analysis.

**Fig. 4:**
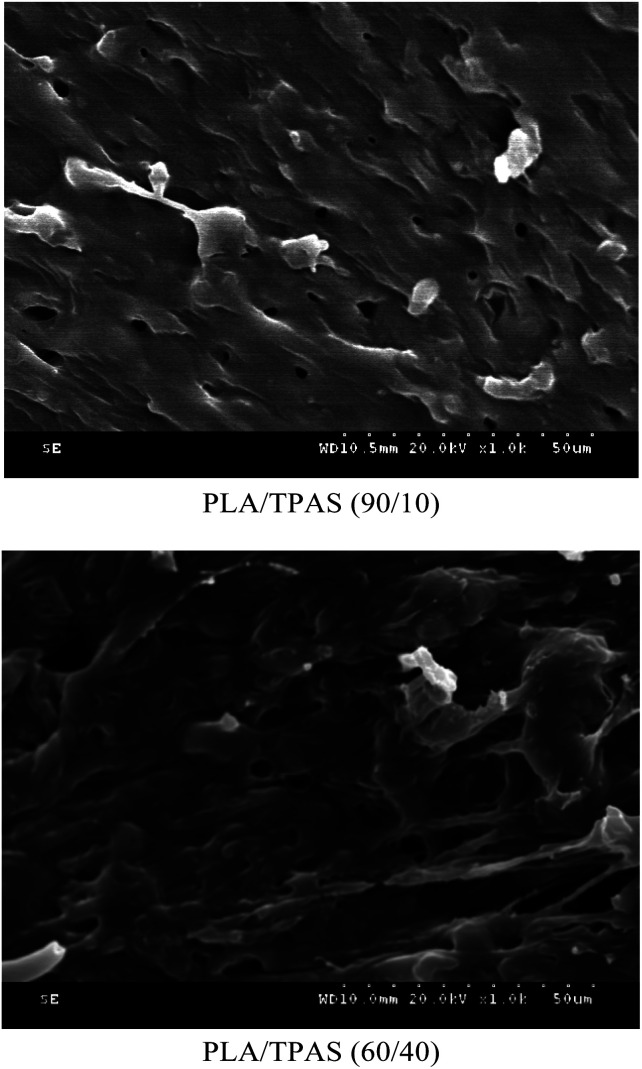
SEM photographs of the PLA/TPAS composites.

Fig. 4 shows the SEM cross-sectional morphology of the PLA/TPAS composite. When the TPAS content is low, such as in PLA/TPAS (90 : 10) (mass ratio) composite materials, TPAS is dispersed in the PLA matrix in the form of particles. There is no obvious interfacial phase between PLA and TPAS, indicating that PLA and TPAS are incompatible. This is consistent with the DSC test results. When the TPAS content increases, such as in PLA/TPAS (60 : 40), there is an interfacial phase between PLA and TPAS. The TPAS particles are almost covered by the matrix PLA. In addition, it can be seen that the size of the dispersed phase is significantly reduced. PLA/TPAS composites exhibit the characteristics of ductile fracture.

### 3.5 TGA analysis of PLA/TPAS

**Fig. 5:**
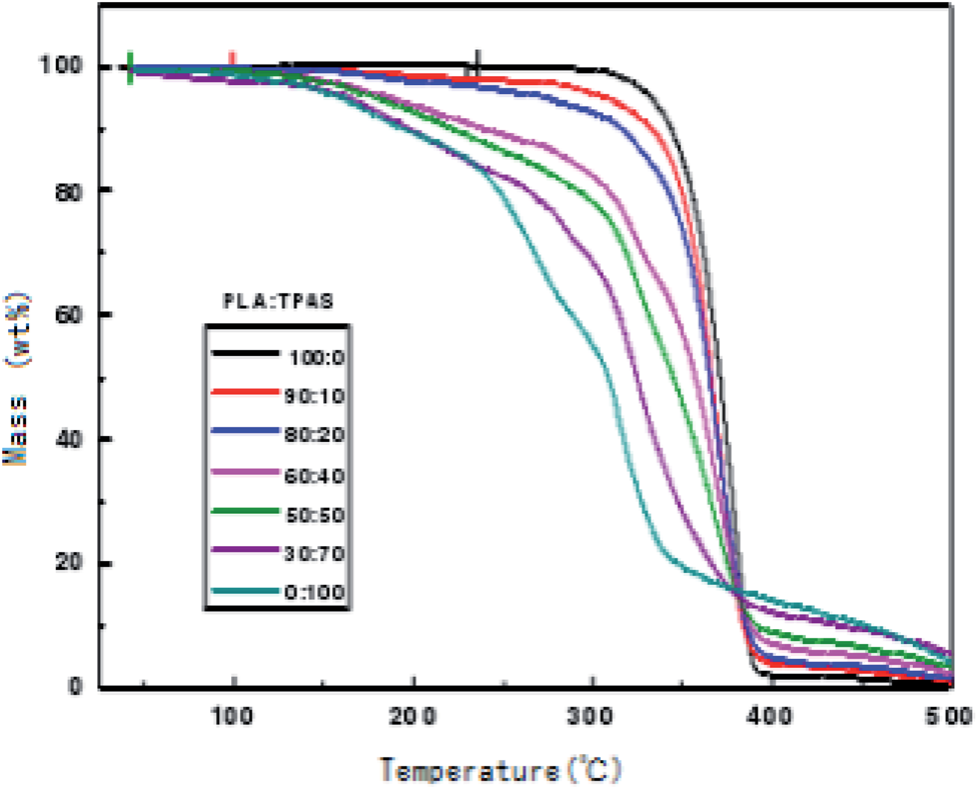
TGA curves of the PLA/TPAS composites.

In order to check the thermal stability of the composite materials, the thermal weight loss of the materials was measured in this work. Fig. 5 shows the TGA curves of the PLA/TPAS composites. It can be seen that under the same experimental conditions, when the weight loss rate is 5%, the thermal weight loss temperature of pure PLA is significantly lower than those of the PLA/TPAS composites. The mass loss of pure TPAS at 100–200 °C is mainly caused by the volatilization of a small amount of the small molecular plasticizers in the system. As the temperature is further increased, the starch in TPAS begins to decompose at around 220 °C. The PLA/TPAS composite materials (0 : 100, 10 : 90, 20 : 80, 40 : 60, 50 : 50, 70 : 30, 100 : 0) show weight loss rates of 0%, 2.1%, 3.6%, and 7.5%, respectively 9.4%, 13.1%, 13.1%. The degradation temperature of pure PLA is around 300 °C, and the weight loss rates of the PLA/TPAS composites are 0.6%, 4.1%, 7.2%, 17.1%, 21.5%, 31.1%, and 44.2%, respectively. With an increase in the TPAS content, the thermal degradation temperature of the system gradually decreases. With the addition of TPAS, the thermal stability of the composite is reduced. However, we also see that the thermal degradation of the composites has a limited effect in the temperature range of the processing of the composites. However, during the processing, a thermal stabilizer should be appropriately added to improve the thermal stability of the material and reduce the impact on its performance.

### 3.6 Biodegradability of PLA/TPAS

#### High temperature and high humidity soil buried degradation

Experiment procedure:

(1) Four plastic foam boxes were prepared, numbered 1, 2, 3, and 4, and 5 cm of humus soil was added to the bottom of these boxes.

(2) Five samples each of pure PLA film, PLA/AS composite film (made by blending PLA with acetylated starch), and PLA/TPAS composite film were prepared, with a sample size of 10 cm × 10 cm. These samples were weighed and the result was recorded as the original weight of the film. The film samples were then spread out on the humus soil, and covered with a further 5 cm of humus soil.

(3) After adding a certain amount of deionized water to the prepared film degradation foam box, it was placed in a constant temperature incubator at 40 °C to carry out degradation experiments. The film degradation foam box was weighed every 12 h and replenished with water. The weight of the film was measured and recorded once every five days (after washing the degraded film with deionized water and weighing and drying it in a vacuum drying oven for 6 h).

**Fig. 6:**
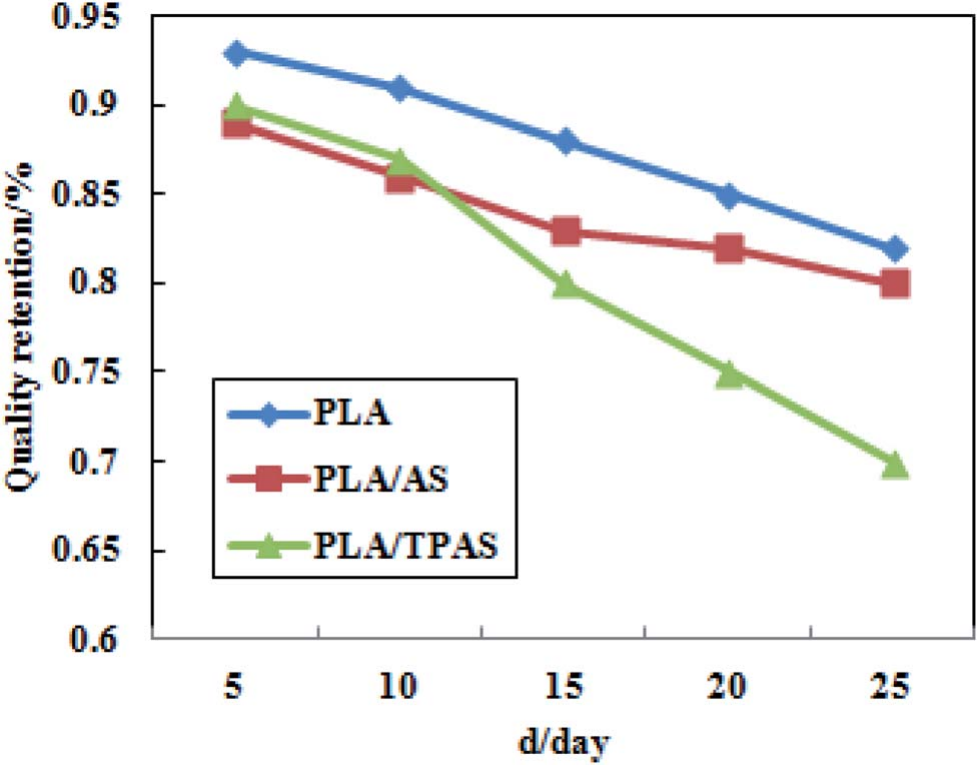
The quality retention after degradation of the PLA-based composite films.

Fig. 6 shows a plot of the mass retention rate of PLA/TPAS after high temperature and high humidity soil degradation. It can be seen from the figure that in the degradation experiments, the degradation rate of pure PLA films was the slowest; followed by the PLA/AS composite films and PLA/TPAS composite films. The reason for the slow degradation rate of the pure PLA film may be that the PLA molecules are closely arranged, blocking the entry of water molecules and making the hydrolysis rate slower. The reason why the PLA/TPAS composite films degrade faster may be that TPAS easily migrates. It migrates into the soil, resulting in the highest rate of mass loss being shown for the composites. The degradation rates of the PLA/TPAS composite films are lower than those of the PLA/AS composite films. The fusion property of TPAS is enhanced, which makes it difficult for small molecules to migrate out, which reduces its mobility. After 15 days of the degradation experiment, the degradation rates of the PLA/TPAS composite films were faster than those of the PLA/AS composite films. This may be caused by the destruction of the original structure of the film, and because the amorphous area of PLA is more likely to be invaded by water molecules, resulting in a faster degradation rate.

#### UV radiation aging experiment

Experimental procedure:

(1) Samples of pure PLA film, and the PLA/AS, PLA/TPAS composite films were cut.

(2) The cut sample strips were placed under a UV lamp tube uniformly, and the strips were sampled every 5 h for tensile performance testing. Five splines were tested for each group of films. Test indicators included the tensile strength and elongation at break. The test results were averaged.

**Fig. 7:**
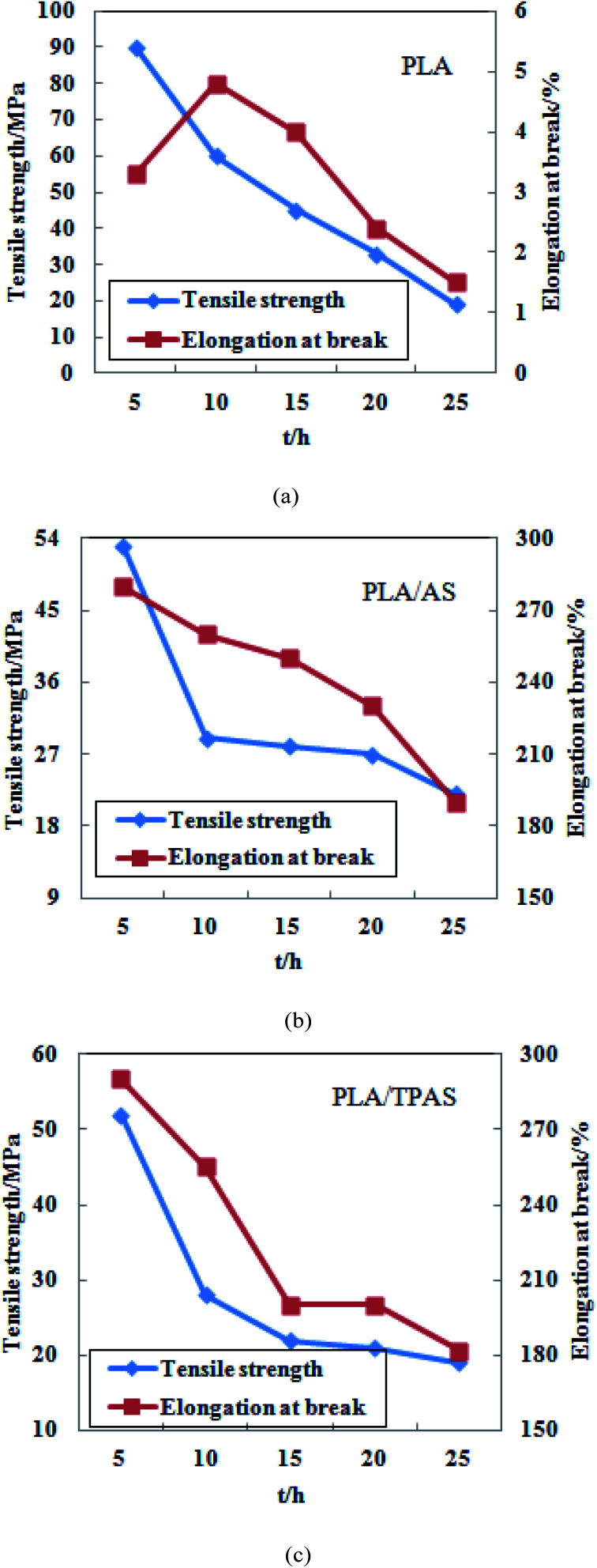
Tensile properties of PLA (a) and PLA/AS (b) PLA/TPAS (c) composite films after UV radiation aging.

Fig. 7 shows the tensile properties of PLA, PLA/AS, PLA/TPAS after UV radiation degradation. The figure shows that the tensile strength and elongation at break of the composite materials both show large changes, indicating that ultraviolet radiation has a significant effect on the tensile properties of the PLA composite films.

Fig. 7(a) shows a graph of the tensile properties of the pure PLA film after UV radiation aging. It can be seen from the figure that the tensile strength of the film reached 90.15 MPa after 5 h of degradation under UV light. After that, as the ultraviolet radiation time increased, the tensile strength of the film continued to decrease. After 25 h, the tensile strength of the film was only 18.32 MPa. After 10 h of UV irradiation, the elongation at break of the film increased. As the irradiation time increased, the elongation at break of the film decreased continuously. After 25 h, the elongation at break of the film was only 1.45%.

Fig. 7(b) shows a graph of the tensile properties of the PLA/AS composite film after UV radiation aging. It can be seen from the figure that as the ultraviolet radiation time increases, the tensile strength and elongation at break of the film decrease continuously. After 5 h of ultraviolet radiation, the tensile strength and elongation at break of the film were 52.24 MPa and 277%, respectively. After 25 h, the tensile strength was 21.67 MPa and the elongation at break was 188%. The tensile strength of the film decreased the most after 10 h of radiation, from 52.24 to 28.96 MPa, after which its decrease slowed.

Fig. 7(c) shows a graph of the tensile properties of the PLA/TPAS composite film after UV radiation aging. It can be seen from the figure that with an increase in the UV radiation time, the tensile strength and elongation at break of the film are similar to those of the PLA/AS composite film. After 5 h of ultraviolet radiation, the tensile strength of the film was 54.60 MPa, and the elongation at break was 294%. After 25 h, the tensile strength was 18.75 MPa and the elongation at break was 182%. After 10 h of irradiation, the tensile strength of the film decreased the most, from 54.60 to 27.06 MPa, and then decreased.

According to the above analysis, the tensile strength of the three films after UV radiation for 5 h increased compared with the tensile strength of the films without radiation experiments, and the increase was greater. The tensile strength of the pure PLA film increased from 62.66 to 90.15 MPa; that of the PLA/AS composite film changed from 32.52 to 52.24 MPa, and that of the PLA/TPAS composite film changed from 35.52 to 54.60 MPa. This shows that UV light irradiation for a proper length of time can cause the PLA molecular chain to rearrange or change the crystal structure, thereby enhancing the tensile strength of the film. During the aging process of materials, ultraviolet light can cause many chemical reactions, resulting in oxidation and degradation of polymer materials. Moreover, these reactions are often chain reactions and are accelerated by the addition of conditions such as temperature, oxygen, and humidity.^42^ The ultraviolet radiation aging experiments were continued on the three films, and it was found that after 10 h of ultraviolet radiation, the tensile strength and elongation at break of the film were significantly reduced. The reason for this may be that the PLA molecular chain is broken under the action of high-intensity ultraviolet light energy. The decrease in the molecular weight of PLA leads to a decrease in its tensile properties.

#### Analysis of degradability in PBS buffer solution

**Fig. 8:**
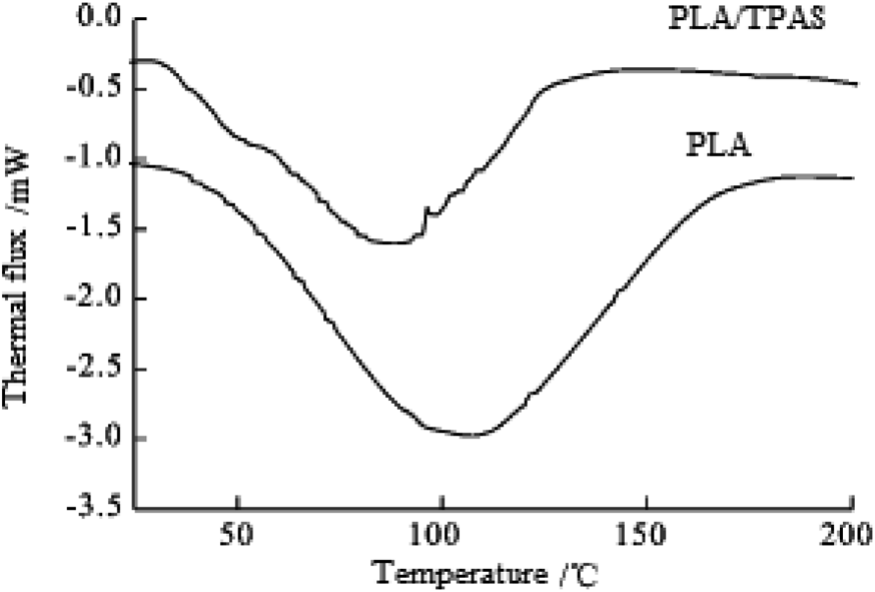
DSC curves of PLA and PLA/TPAS.

Degradation experiments were performed on PLA and PLA/TPAS, and the degradation with temperature in PBS buffer solution at pH 7.4 was compared. It can be seen from Fig. 8 that under the same conditions, the degradation rate of PLA/TPAS is faster than that of PLA. There are two stages of PLA degradation. In the first stage, water molecules diffuse into the amorphous region of the PLA, and the C–O ester bonds in the molecular chain are randomly broken. The undegraded segments gain more space and activity, and the crystallinity of the molecular chain rearrangement is improved. In the second stage, when the degradation of the amorphous region is almost over, the hydrolysis starts from the edge of the crystalline region and expands toward the crystalline center, but the speed is much slower than that of the amorphous region.

The degradation of PLA/TPAS can also be divided into two stages. In the first stage, PLA/TPAS is rapidly degraded. This is because the acid anhydride bond and the remaining hydroxyl groups in the composite material destroy the crystallinity of PLA, and also enhance the ability of water molecules to attack ester bonds and hydrophilic groups, resulting in accelerated hydrolysis. Therefore, the degradation rate of the composite material increases. At the most, two thirds of the hydroxyl groups in the PLA molecules undergo graft copolymerization. The lower the degree of hydroxyl substitution, the more hydrophilic the copolymer and the faster the degradation rate. Conversely, the higher the grafting rate, the less starch components in the copolymer, and the slower the degradation rate. During the degradation process, the PLA/TPAS macromolecule chains are gradually broken into small molecules. They diffuse from the sample surface or from the inside of the sample to the surface *via* molecular motion and dissolve in the degradation medium. In the second stage, PLA/TPAS is slowly degraded. After 100 °C, the degradation rate slows down. Most of the easily degradable hydrophilic backbone molecules have been degraded. The side chain PLA is broken into small segments, in the same way as for PLA. It can be seen that the introduction of TPAS increases the degradation rate of PLA molecules.

## 4 Conclusion

Adding modified starch TPAS can effectively improve the mechanical properties of PLA. The elongation at break of the composites increased with increasing TPAS content. When the amount of TPAS added was 40% by weight, the elongation at break increased by more than 4 times. During the stretching process, the composites exhibited significant ductile fracture. The tensile strength and modulus gradually decreased with an increase in the TPAS content. When the TPAS content is 40 wt%, the mechanical properties of the PLA/TPAS composites are the best. DSC and DMA analysis shows that the PLA/TPAS composites are incompatible systems. The addition of TPAS promotes the crystallization of PLA and reduces the thermal stability of the material. However, during the material processing, its degradation behavior is limited, which has little effect on the performance of the material.

In the PLA/TPAS composite high-temperature and high-humidity soil buried degradation experiment, the degradation rate of the pure PLA film was the slowest. In the 25 day degradation experiment, PLA showed the highest mass retention rate. The modified PLA-based composite film showed the fastest degradation rate and the lowest mass retention rate. This shows that the addition of AS and TPAS destroys the original molecular chain structure of PLA and accelerates its degradation rate. In the ultraviolet radiation degradation experiment, the tensile strength of the composite film was improved in the first few hours. This may be caused by the composite film being crosslinked or entangled, or its crystal structure changing under high energy irradiation UV light. After that, with an increase in the ultraviolet radiation time, the tensile properties of the composite films were significantly reduced. The degradability analysis in PBS buffer solution showed that under the same conditions, the degradation rate of PLA/TPAS was faster than that of PLA.

## Conflicts of interest

There are no conflicts to declare.

## Supplementary Material
